# Cytoophidia Influence Cell Cycle and Size in *Schizosaccharomyces pombe*

**DOI:** 10.3390/ijms25010608

**Published:** 2024-01-03

**Authors:** Ruolan Deng, Yi-Lan Li, Ji-Long Liu

**Affiliations:** 1 School of Life Science and Technology, ShanghaiTech University, Shanghai 201210, China; 2 CAS Center for Excellence in Molecular Cell Science, Shanghai Institute of Biochemistry and Cell Biology, Chinese Academy of Sciences, Shanghai 200031, China; 3 University of Chinese Academy of Sciences, Beijing 100049, China; 4 Department of Physiology, Anatomy and Genetics, University of Oxford, Oxford OX1 3PT, UK

**Keywords:** cell cycle, cell size, CTP synthase, cytoophidium, G2 phase, *Schizosaccharomyces pombe*, slm9

## Abstract

Cytidine triphosphate synthase (CTPS) forms cytoophidia in all three domains of life. Here we focus on the function of cytoophidia in cell proliferation using *Schizosaccharomyces pombe* as a model system. We find that converting His^359^ of CTPS into Ala^359^ leads to cytoophidium disassembly. By reducing the level of CTPS protein or specific mutation, the loss of cytoophidia prolongs the G2 phase and expands cell size. In addition, the loss-filament mutant of CTPS leads to a decrease in the expression of genes related to G2/M transition and cell growth, including histone chaperone *slm9*. The overexpression of *slm9* alleviates the G2 phase elongation and cell size enlargement induced by CTPS loss-filament mutants. Overall, our results connect cytoophidia with cell cycle and cell size control in *Schizosaccharomyces pombe*.

## 1. Introduction

The nucleotide Cytidine triphosphate (CTP) is a rate-limiting enzyme that catalyzes the ATP-dependent conversion of UTP to CTP [1], which plays a crucial role in various biological processes, including DNA and RNA synthesis, phospholipid synthesis, protein glycosylation, and energy transfer enzymatic reactions across diverse organisms. Notably, CTPS has been observed to form filamentous structures, termed cytoophidia, in a wide range of organisms from prokaryotes to eukaryotes. CTPS cytoophidium was first identified in *Drosophila*, *Caulobacter crescentus*, and *S. cerevisiae* in 2010 [2,3,4], with subsequent studies confirming its presence in human cells [5,6], fission yeast [7], plants [8], archaea [9], and mammalian cells [10]. These findings highlight the highly conserved nature of cytoophidia among different organisms and suggest that they possess significant yet undiscovered biological functions.

The role of cytoophidia remains enigmatic despite proposed physiological functions in various organisms. Multiple studies have demonstrated that cytoophidia regulate cell metabolism by adopting different conformations to control CTPS enzymatic activity across different organisms [11,12,13,14,15,16]. In *C. crescentus*, cytoophidia cooperated with the intermediate filaments to maintain cellular shape [4]. In *Drosophila*, cytoophidia are involved in optic lobe development and oogenesis; recent studies showed that cytoophidia take part in adipose architecture and metabolism, and loss of cytoophidia reduced adipocyte expansion and inhibition of lipogenesis [15,17,18,19]. In human cells, cytoophidia stabilize the CTPS protein by extending its half-life [20,21] and abnormal cytoophidia affects cell survival [22]. Cytoophidia participate in ovarian stress response in *Drosophila* [23] and respond to changes in nutrition, pH, temperature, and osmolality for stress adaptation in yeast [24,25]. Furthermore, cytoophidia contributed to rapid cell proliferation, and they have been observed in neuroblasts of *Drosophila* larvae [13] and highly proliferated T cells within the murine thymus [10]. Additionally, elevated CTPS activity has been found in various cancers such as hepatoma and lymphoma; moreover, cytoophidia have been identified within human cancers like hepatocellular carcinoma [5,26,27], suggesting an important role for cytoophidia in cell proliferation. 

The cytoophidia are believed to play a crucial role in coupling cell proliferation, particularly in fast-growing cells like cancer cells and activated T cells [5,10]. Notably, studies have shown that mouse embryonic stem cells possess abundant cytoophidia [28]. In the context of cancer, the formation of cytoophidia may indicate an abnormal capacity for rapid proliferation. However, none of these studies explicitly elucidate the specific function that cytoophidia undertake in cell proliferation. The absence of CTPS redundancy provides *S. pombe* with a distinct advantage as a model organism for investigating the function of cytoophidia. In comparison to humans and *S. cerevisiae*, which possess two CTPS isoforms (CTPS1 and CTPS2 in humans; Ura7 and Ura8 in *S. cerevisiae*), *S. pombe* only harbors one CTPS isoform encoded by a single locus *cts1* on chromosome I.

In this study, we utilize *S. pombe* as a model system to investigate the role of CTPS cytoophidia in cellular proliferation. Our results demonstrate that the absence of cytoophidia leads to a delay in the G2 phase of the cell cycle and an increase in cell size. Interestingly, there exists a mutual dependence between cytoophidia and CTPS protein levels; specifically, loss of cytoophidia decreases protein levels while protein levels influence cytoophidia formation. The expression levels of multiple genes associated with cell growth and proliferation are reduced in strains lacking cytoophidia. Overexpression of Slm9, a histone transcription regulator involved in regulating Cdc2 via Wee1, mitigates the prolongation of the cell cycle caused by loss-filament mutants of Cts1. These findings provide valuable insights into the regulatory roles played by cytoophidia during cellular proliferation.

## 2. Results

### 2.1. Cytoophidia Are Observed Exclusively during the Log Phase of S. pombe

**Figure 1:**
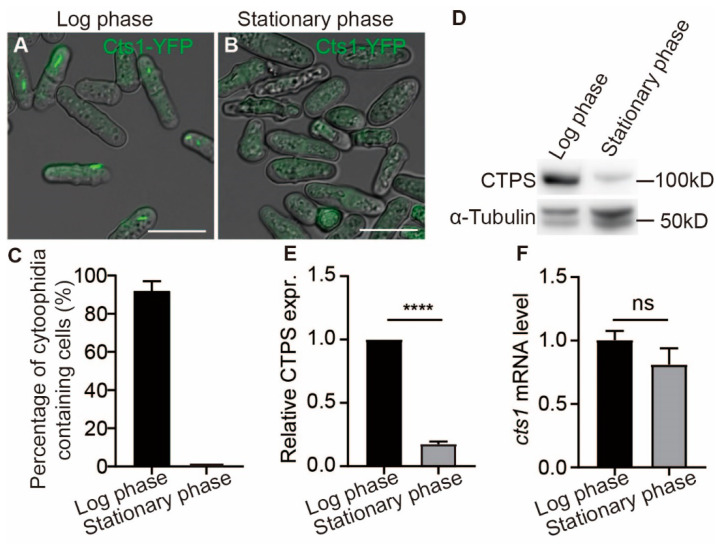
Cytoophidia present only in the log phase of *S. pombe*. CTP synthase (CTPS) fused to YFP was expressed under the endogenous promoter. (**A**) The CTPS protein form the cytoophidia at log phase in fission yeast. (**B**) Cytoophidia not present in stationary phase. Scale bars, 10 μm. (**C**) percentage of cells containing cytoophidia in fission yeast at log phase and stationary phase. (**D**) Western blotting analysis of CTPS proteins from Cts1-YFP tagged fission yeast at log and stationary phase. Anti-YFP antibody was used. Alpha-tubulin was used as an internal control. (**E**) Quantification of the CTPS protein relative expression levels in (**D**) (3 biological replicates). (**F**) Levels of CTPS mRNA expression at log and stationary phase, detected by real-time PCR. Values were normalized against 18s rRNA expression (3 biological replicates). All values are mean ± S.E.M. ****, *p* < 0.0001; ns, no significant difference by Student’s *t*-test.

The growth of *S. pombe* cells can be divided into four distinct stages: lag phase, logarithmic phase, stationary phase, and death phase. Each phase is characterized by unique metabolic conditions. We previously reported that cytoophidia are regulated by the metabolic conditions within *S. pombe* [29]. Utilizing the Cts1-YFP tagged strain previously established in our laboratory, we observed a high abundance of cytoophidia during the logarithmic phase: cytoophidia were present in over 90% of fission yeast cells (Figure 1A,C) and subsequently disappeared during the stationary phase (Figure 1B,C). Furthermore, compared to the logarithmic phase, there was a decrease in CTPS protein levels during the stationary phase while CTPS RNA levels remained unchanged (Figure 1D–F, Supplemental Figure S5). These findings indicate that cytoophidia are exclusively present during the logarithmic growth stage of fission yeast and suggest their potential importance in cell proliferation.

### 2.2. Loss-Filament Mutation Cts1-H359A Impacts the Cell Cycle and Size of S. pombe

**Figure 2:**
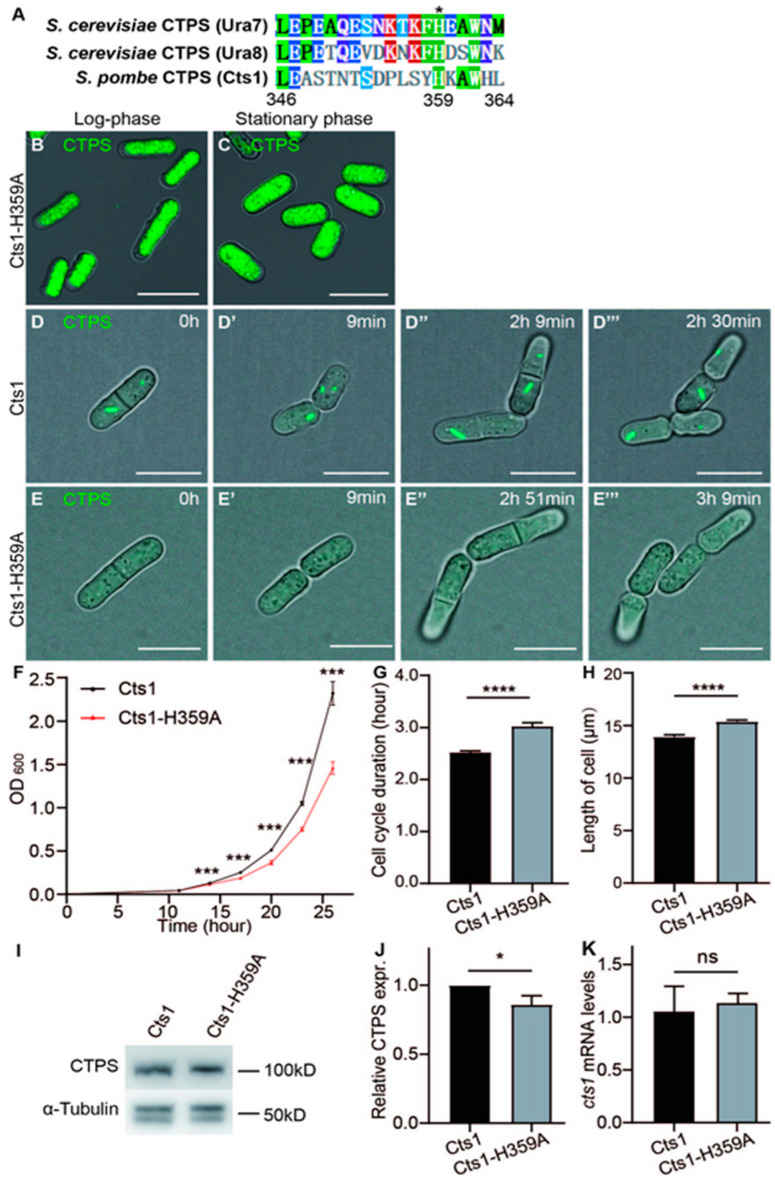
Loss-filament mutation Cts1-H359A affects the cell cycle and size in *S. pombe*. CTP synthase (CTPS) fused to YFP was expressed under the endogenous promoter. (**A**) The alignment of CTPS amino acids from *S. cerevisiae* (Ura7 and Ura8) and *S. pombe* (Cts1). Conserved amino acids (aa) are in colors. Aliphatic aa leucine (L), proline (P), alanine (A), methionine (M) in green; Aromatic aa phenylalanine (F), tryptophan (W) and histidine (H) in grass green; acidic aa glutamate (E) in blue; amidic aa glutamine (Q) and asparagine (N) in indigo; hydroxylic aa serine (S) and threonine (T) in skyblue; basic aa lysine (K) in red. Non-conserved aa are colored in white background. (**B**,**C**) Images of Cts1-H359A strain at log phase (**B**) and stationary phase (**C**). Channels: green (CTPS) and bright field. Scale bars, 10 μm. (**D**–**E’’’**) Live-cell imaging of Cts1 strain (**D**–**D’’’**) and Cts1-H359Astrain (**E**–**E’’’**) at the log phase. Channels: green (CTPS) and bright field. Scale bars, 10 μm. (**F**) Growth curves of Cts1 and Cts1-H359Astrains (3 biological replicates). (**G**,**H**) Quantifications of cell cycle duration (**G**) and cell length (**H**) obtained from live imaging (>86 cells were manually measured per strain) (3 biological replicates). (**I**) Western blotting analysis of CTPS proteins from Cts1 and Cts1-H359A strains at log phase. Anti-YFP antibody was used. Alpha-tubulin was used as an internal control. (**J**) Quantification of the CTPS protein relative expression levels in I (3 biological replicates). (**K**) CTPS mRNA expression level detected by real-time PCR at log phase. Values were normalized against 18s rRNA expression (3 biological replicates). All values are mean ± S.E.M. **** *p* < 0.0001, *** *p* < 0.001, * *p* < 0.05; ns, no significant difference by Student’s *t*-test.

To investigate the role of cytoophidia in fission yeast, we specifically disrupted the polymerization of CTPS to form cytoophidia without affecting their enzymatic functions. Previous studies have demonstrated that the histidine amino acid at position 355 (His^355^) in human and *Drosophila*, as well as His^360^ in *S. cerevisiae*, is crucial for CTPS polymerization [18,24]. The alignment of CTPS protein sequences from *S. cerevisiae* (Ura7 and Ura8) and *S. pombe* (Cts1) shows the conserved His (labelled as No. 359) in all three species (Figure 2A). Therefore, we hypothesized that the replacement of His^359^ with Ala^359^, an uncharged and smaller amino acid compared to histidine, would disrupt the polymerization ability of the *S*. *pombe* CTPS protein like that observed in humans, *Drosophila*, and *S. cerevisiae*. 

Consequently, we generated a mutation where His^359^ was replaced by Ala^359^ (referred to as H359A) within the endogenous CTPS protein of *S. pombe*. Additionally, we fused YFP at the C-terminus of the mutant CTPS for easy detection (Cts1-H359A-YFP). By extracting genomic DNA from the Cts1-H359A starin, we confirmed successful introduction of the intended mutation (Supplemental Figure S1). The CTPS H359A mutant fission yeast was viable but did not form cytoophidia (Figure 2B,C). 

Furthermore, measurement via the OD_600_ value showed that the Cts1-H359A strain exhibited a slower growth rate compared to the Cts1-YFP strain which served as the control group (Figure 2F). Live-cell imaging data showed that the Cts1-H359A strain had an extended cell cycle and increased cell length in comparison to the control (Figure 2D–E’’’,G,H). Interestingly, Western blotting analysis indicated reduced levels of CTPS protein in the H359A mutant strain when compared with the control strain (Figure 2I,J, Supplemental Figure S5), while real-time PCR showed similar levels of CTPS RNA between them (Figure 2K). 

Combined with the data of our previous studies [18,20], these findings suggest that cytoophidia formation is crucial for maintaining adequate levels of CTPS protein in *S. pombe*, potentially through polymerization to ensure protein stability; they also indicate that failure to form CTPS cytoophidia leads to elongated cell cycles and influences cell size.

### 2.3. Both CTPS Protein Levels and Cytoophidia Affect the Cell Cycle and Size of S. pombe

**Figure 3:**
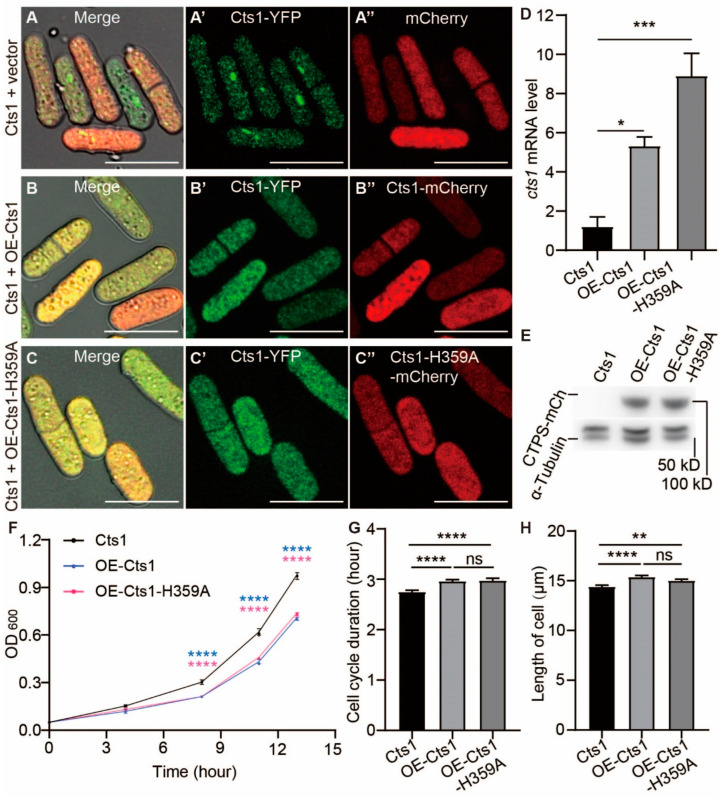
CTPS protein levels influence cytoophidia formation, cell cycle, and size in *S. pombe*. CTP synthase (CTPS) fused to YFP was expressed under the endogenous promoter. (**A**–**C’’**) Images of CTPS-YFP strains that overexpressed mCherry (**A**–**A’’**), Cts1-mCherry (**B**–**B’’**), and Cts1-H359A-mCherry (**C**–**C’’**) at the log phase. Channels: green (CTPS), red (mCherry), and bright field. Scale bars, 10 μm. (**D**) Levels of CTPS mRNA expression as detected by real-time PCR in control (Cts1), OE-Cts1, and OE-Cts1-H359A strains at log phase. Values were normalized against 18s rRNA expression. One-way ANOVA was used for statistical analysis. (**E**) Western blotting analysis of CTPS proteins from control (Cts1), OE-Cts1, and OE-Cts1-H359A strains at log phase. Anti-mCherry antibody was used. Alpha-tubulin was used as an internal control. (**F**) Growth curve of control (Cts1), OE-Cts1, and OE-Cts1-H359A strains. Unpaired Student’s *t*-test was used for statistical analysis (3 biological replicates). (**G**,**H**) The cell cycle duration (**G**) and cell length (**H**) obtained from the data of live-cell imaging (>85 cells were manually measured per strain). One-way ANOVA was used for statistical analysis (2 biological replicates). All values are mean ± S.E.M. **** *p* < 0.0001, *** *p* < 0.001, ** *p* < 0.01, * *p* < 0.05; ns, no significant difference.

To understand whether the cytoophidia and the CTPS protein levels influenced the cell cycle and size, we firstly disrupted filament formation by creating an OE-Cts1-H359A strain that overexpressed a mutant, CTPS (H359A), tagged with mCherry at its C-terminus. Thus, we can insulate the effect of cytoophidium formation from protein level on cell cycle and size. Then, we generated an OE-Cts1 strain that overexpressed wild-type CTPS tagged with mCherry as well as a strain overexpressing only mCherry as the controls. The strains were confirmed by real-time PCR and Western blotting (Figure 3D,E, Supplemental Figure S5). 

In both the log and stationary phases, the presence of cytoophidia was not observed in either OE-Cts1 or the OE-Cts1-H359A strain, suggesting that the H359A mutation can act dominantly to inhibit cytoophidia formation (Figure 3C–C’’ and Supplemental Figure S3C–C’’). Surprisingly, even in the presence of overexpressed wild-type CTPS in the OE-Cts1 strain, cytoophidia did not appear during both log phase (Figure 3A–B’’) and stationary phase (Supplemental Figure S2A–B’’). Additionally, based on OD_600_ measurements from live curves, both the OE-Cts1 and OE-Cts1-H359A strains grow slower than the control (Figure 3F). 

Live-cell imaging data revealed that both these strains had longer cell cycles and increased cell length compared to the control (Figure 3G,H). These observations were consistent with those seen in the Cts1-H359A strain (Figure 2G–H), indicating an influence of both cytoophidia formation and CTPS protein levels on cell proliferation rate.

**Figure 4:**
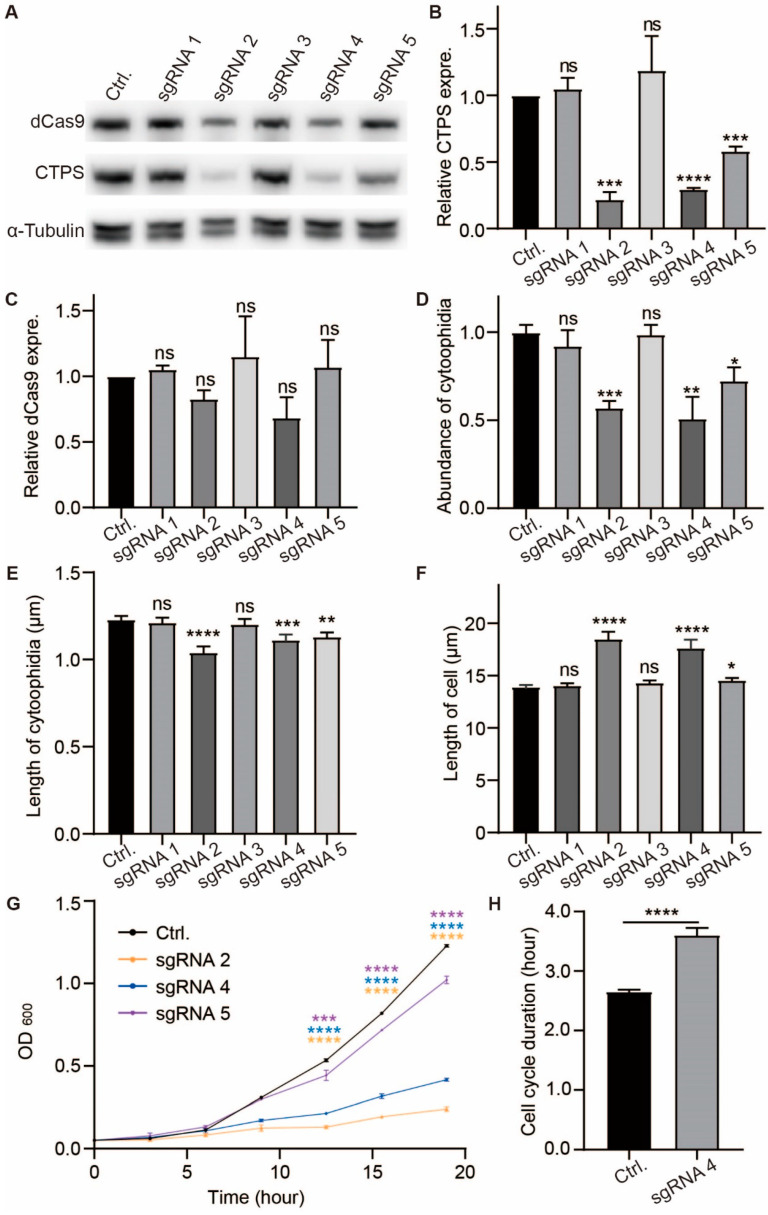
CTPS reduction affects cytoophidia formation, elongates cell cycle, and increases cell size. (**A**) Immunoblotting analysis of CTPS and dCas9 proteins from the Cts1-YFP strains which expressed the pdCas9 plasmid carried nonsense sgRNA (as control group), sgRNA1, sgRNAs2, sgRAN3, sgRAN4, and sgRAN5, respectively, at log phase. Anti-YFP and anti-flag antibodies were used. Alpha-tubulin was used as an internal control. (**B**,**C**) Quantification of the CTPS protein (**B**) and dCas9 protein (**C**) relative expression levels in A (3 biological replicates). (**D**–**F**) Quantification of the numbers of cytoophidia (**D**), length of cytoophidia (**E**), and cell length (**F**) in the strains which contained sgRNA and dCas9. The relative value was normalized with control (>265 cells were manually measured, 2 biological replicates). (**G**) The growth curve of the strains contained nonsense sgRNA (as control group), sgRNA2, sgRNAs4, and sgRAN5 (3 biological replicates). (**H**) The cell cycle duration obtained from the data of live-cell imaging (>230 cells were manually measured, 2 biological replicates). All values are the mean ± S.E.M. **** *p* < 0.0001, *** *p* < 0.001, ** *p* < 0.01, * *p* < 0.05; ns, no significant difference by Student’s *t*-test.

Second, to investigate whether the decreasing CTPS protein levels influenced the formation of cytoophidia and cell proliferation, we knocked down the CTPS using the CRISPRi method based on the CRISPR-dCas9 system [30]. The expression of dCas9 protein was regulated by the nmt1-41 promoter, which induced transcription upon thiamine removal from the medium (Supplemental Figure S3A). Five sgRNAs were designed to target the *cts1* gene encoding CTPS protein in fission yeast (Supplemental Figure S3B). Each of these sgRNAs was transformed into a Cts1-YFP tagged fission yeast strain, while a non-homologous sgRNA-containing strain served as a control group. Western blotting analysis confirmed the expression of dCas9 protein in all strains (Figure 4A,C, Supplemental Figure S5) and showed that CTPS protein levels were reduced in strains containing sgRNA2, sgRNA4, and sgRNA5 compared to the control group after thiamine removal (Figure 4A,B, Supplemental Figure S5). However, no significant changes in CTPS protein levels were observed in cells containing sgRNA1 and sgRNA3 (Figure 4A,B, Supplemental Figure S5). 

SgRNA2, sgRNA4, and sgRNA5 successfully knocked down CTPS and resulted in decreased abundance and length of cytoophidia when compared with the control group, whereas no changes were found in cells with sgRNA1 or sgRNA3 (Figure 4D,E, Supplemental Figure S3C–H). Measurement via OD_600_ values revealed slower cell growth rates for CTPS knockdown strains (sgRNA2, sgRNA4, and sgrNA5) compared to the control group (Figure 4G). The growth rate of sgRNA-targeted strains was sgRNA5 > sgRNA4 > sgRNA2, which the CTPS protein knockdown levels increased sequentially (Figure 4G), and the cell length of these strains was also longer than the control (Figure 4F). Furthermore, live curve data indicated an extended cell cycle duration for CTPS knockdown cells compared to control (Figure 4H). 

The findings demonstrate that the modulation of CTPS protein levels has an impact on cytoophidia formation and cellular proliferation. Moreover, both CTPS protein levels and cytoophidia in *S. pombe* exert an influence on cell cycle progression and size regulation.

### 2.4. Cts1-H359A Mutation Extends the Duration of the G2 Phase in S. pombe

The Cts1-H359A strain exhibited a longer cell cycle duration compared to the Cts1 strain. Therefore, we aimed to investigate which specific phase of the cell cycle (S, M, G1, or G2) was affected by this mutation. In fission yeast, the nuclear cell cycle is divided into distinct phases: G1, S, G2, and M. Following mitosis, newly replicated nuclei enter the subsequent cell cycle and undergo G1 and S phases before completing cytokinesis from the previous cycle [31,32]. This differs from mitosis in mammals and budding yeast. 

**Figure 5:**
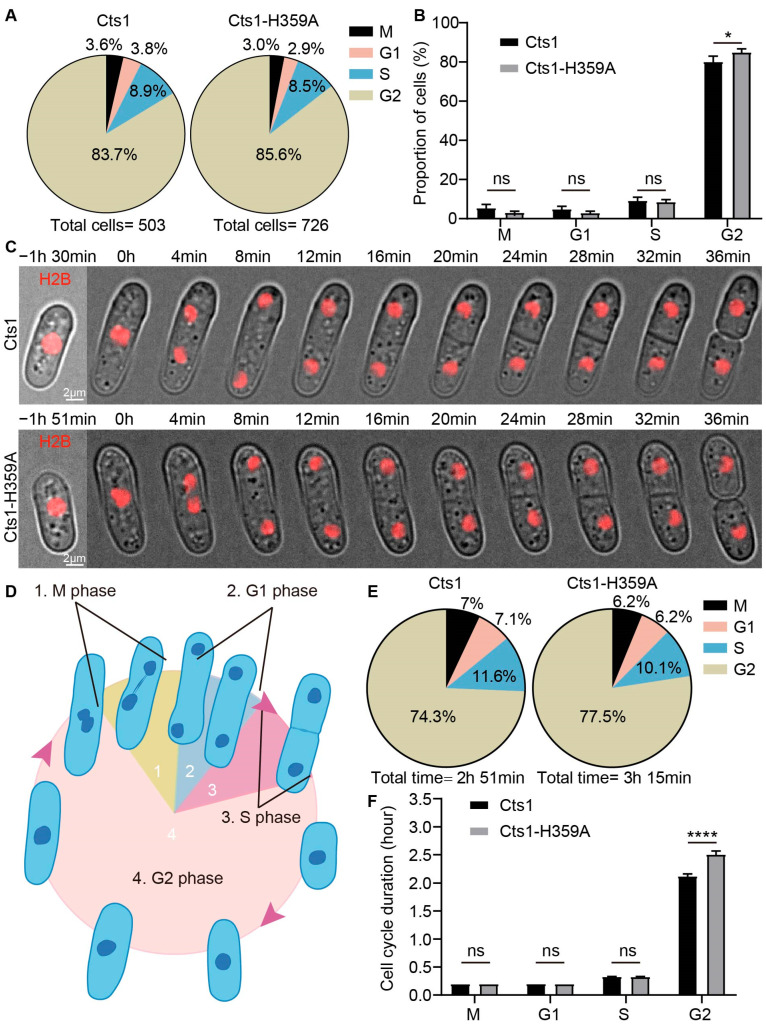
Cts1-H359A mutation prolongs the G2 phase in *S. pombe*. (**A**) The proportion of cells in M, G1, S, and G2 phase of the whole cell cycle, from the fission yeast that was treated with hydroxyurea (HU). (**B**) The proportion of cells in M, G1, S, and G2 phase in (**A**). (**C**) Live-cell imaging photos of H2B-mCherry-expressed Cts1 and Cts1-H359A strains. Channels: red (H2B) and bright field. Scale bars, 2 μm. (**D**) Partitioning diagram of M, G1, S, and G2 phase during fission yeast mitosis. (**E**) The time proportion of M, G1, S, and G2 phase in the whole cell cycle from the data of live-cell imaging; >100 cells were manually measured per strain. (**F**) The cell cycle durations of M, G1, S, and G2 phase between Cts1 and Cts1-H359A strains. All values are means ± S.E.M. **** *p* < 0.0001, * *p* < 0.05; ns, no significant difference by Student’s *t*-test.

First, we used hydroxyurea (HU) to induce cell cycle synchronization at the beginning of the S phase; the fission yeast was treated with HU for 4 h followed by a release period of 3 h. Based on the literature summarizing mitosis in fission yeast (Figure 5D), we performed microscopic imaging of HU-treated cells and stained their nuclei with DAPI dye. Analyzing the state and morphology of the nucleus in these images and statistically evaluating data (Figure 5A), we observed an increased proportion of cells in the G2 phase within the Cts1-H359A strain compared to the Cts1 strain (Figure 5B). The findings suggest that there is a prolonged duration of the G2 phase during cell cycle progression in the Cts1-H359A strain.

To validate the aforementioned inference, we conducted live-cell imaging to observe cell division in fission yeast. To label the nucleus, H2B-mCherry was expressed in both the Cts1-H359A strain and Cts1 strain. Our results showed that H2B-mCherry was observed in both strains (Figure 5C). 

Following an analysis of the live-cell imaging data and observation of nuclear state and morphology, we quantified the proportions of G1, S, G2, and M phase durations in the cell cycle (Figure 5E). Compared with Cts1 strain, the G2 phase was prolonged in the Cts1-H359A strain, while no significant differences were observed for the G1, S, and M phases (Figure 5F). These findings indicate that Cts1-H359A mutation could extend the G2 phase.

### 2.5. Cts1-H359A Mutation Perturbs G2/M Transition and the Expression of Cell Growth-Related Genes

**Figure 6:**
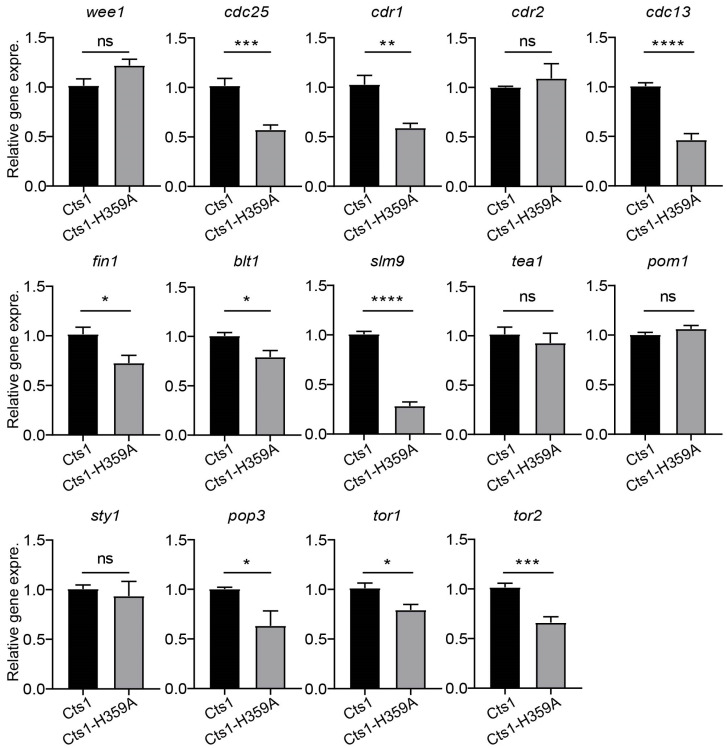
Loss-filament mutation of CTPS perturbs G2/M transition and the expression of cell growth-related genes. RNA levels of some regulators of cell size and cell cycle in G2/M transition in CTPS^H359A^-YFP and CTPS-YFP strains at log phase detected by real-time PCR. Including G2/M transition regulators: *wee1*, *cdc25*, *cdr1*, *cdr2*, *cdc13*, *fin1*, *blt1*, and *slm9*; cell growth regulators: *tea1*, *pom1*, *sty1*, *pop3*, *tor1*, and *tor2*. All values are means ± S.E.M. **** *p* < 0.0001, *** *p* < 0.001, ** *p* < 0.01, * *p* < 0.05; ns, no significant difference by Student’s *t*-test (3 biological replicates).

To investigate whether Cts1-H359A mutation affects the expression of genes related to cell growth and G2/M transition, we assessed the expression levels of relevant genes using real-time PCR in fission yeast at the logarithmic phase, including G2/M transition regulators (*wee1*, *cdc25*, *cdr1*, *cdr2*, *cdc13*, *fin1*, *blt1*, and *slm9*) and cell growth regulators (*tea1*, *pom1*, *sty1*, *pop3*, *tor1*, and *tor2*). Our data revealed that in the Cts1-H359A strain, the gene expression levels of *cdc25*, *cdr1*, *cdc13*, *fin1*, *blt1*, *slm9*, *pop3*, *tor1*, and *tor2* were decreased compared with those in the Cts1 strain (Figure 6). This suggests that Cts1-H359A mutation influences the expression of genes associated with cell growth and G2/M transition.

### 2.6. Overexpression of slm9 Alleviates Phenotypes Caused by Cts1-H359A Mutation

The histone transcription regulator Slm9 has been previously shown to regulate Cdc2 activity through Wee1, and Slm9 deficiency strains exhibit a G2 cell cycle delay [33]. Immunoaffinity purifications of Slm9 have revealed the co-purification of Cts1 with Slm9 proteins [34]. Additionally, our previous study reported that *slm9* knockout affects cytoophidia, increases cell volume, and slows down cell growth in *S. pombe* [35]. 

**Figure 7:**
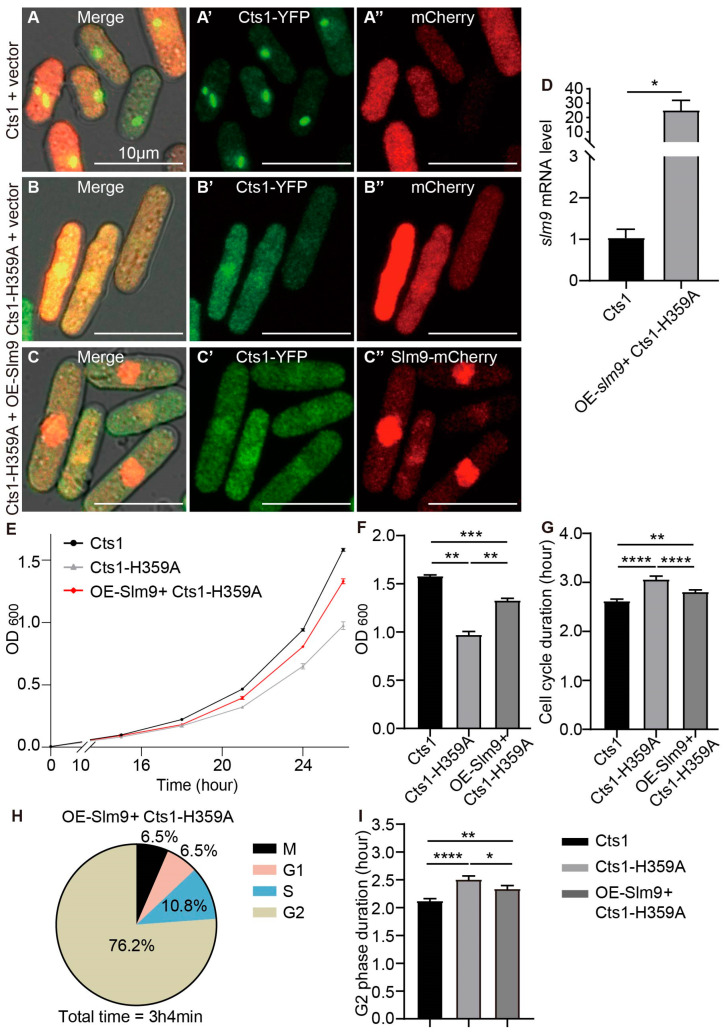
Overexpressing *slm9* alleviates cell cycle prolongation caused by Cts1-H359A mutation. CTP synthase (CTPS) fused to YFP was expressed under the endogenous promoter. (**A**–**C’’**) Images of Cts1 strain that overexpressed mCherry (**A**–**A’’**), Cts1-H359A strain that overexpressed y mCherry (**B**–**B’’**), and Cts1-H359A strain that overexpressed Slm9-mCherry (**C**–**C’’**) at the log phase. Channels: green (CTPS), red (mCherry), and bright field. Scale bars, 10 μm. (**D**) *slm9* expression levels at log phase detected by qPCR. Values were normalized against 18s rRNA expression. Unpaired Student’s *t*-test was used for statistical analysis. (**E**) Growth curve of Cts1, Cts1-H359A, and OE-Slm9+ Cts1-H359A strains (3 biological replicates). (**F**) The OD_600_ value of Cts1, Cts1-H359A, and OE-Slm9+ Cts1-H359A strains at 26 h in (**E**); statistical analysis by one-way ANOVA. (**G**) The cell cycle durations obtained from the data of live-cell imaging (>120 cells were manually measured per strain). One-way ANOVA was used for statistical analysis. (**H**) The proportions of the duration of M, G1, S, and G2 in the whole cell cycle from the live-cell image data of OE-Slm9+ Cts1-H359A strain (>100 cells were manually measured per strain). (**I**) The G2 phase duration of the Cts1, Cts1-H359A, and OE-Slm9+ Cts1-H359A strains. Statistical analysis by one-way ANOVA (>100 cells were manually measured per strain). All values are the mean ± S.E.M. **** *p* < 0.0001, *** *p* < 0.001, ** *p* < 0.01, * *p* < 0.05.

The reduced *slm9* expression level in the Cts1-H359A strain suggests that Slm9 is involved in G2 phase prolongation caused by Cts1-H359A mutation. Therefore, we investigated whether overexpressing *slm9* could rescue prolonged cell cycles in the Cts1-H359A strain. We overexpressed *slm9*-mCherry in the Cts1-H359A strain. Slm9 is localized to the nucleus (Figure 7C–C’’, Supplemental Figure S4). In addition, we overexpressed only mCherry in the Cts1 and Cts1-H359A strains, respectively, as the control strains (Figure 7A–B’’, Supplemental Figure S4). The *slm9* expression levels in the OE-Slm9+ Cts1-H359A strain were confirmed by real-time PCR (Figure 7D). 

Growth curves measured via OD600 values showed that the OE-Slm9+ Cts1-H359A strain grew faster than the Cts1-H359A strain but slower than the Cts1 strain (Figure 7E). A statistically significant difference was observed at 26 h when comparing OD600 values between these strains, indicating that OE-Slm9+ Cts1-H359A grows faster than loss-filament mutants but slower than normal unmutated strains (Figure 7F). Furthermore, analysis of live-cell imaging data revealed that the cell cycle duration of the OE-Slm9+ Cts1-H359A strain was shorter than that of the Cts1-H359A strain but longer than that of the Cts1 strain (Figure 7G). Additionally, a statistical analysis was performed on the proportion of duration for G1, S, G2, and M phases throughout the entire cell cycle of the OE-Slm9+ Cts1-H359A strain (Figure 7H,I). 

Comparisons of the G2 phase duration among the Cts1, Cts1-H359A, and OE-Slm9+ Cts1-H359A strains revealed that in the OE-Slm9+ Cts1-H359A strain, the G2 phase was shorter compared with that in the Cts1-H359A strain but longer than in the Cts1 strain. These results indicated that overexpression of *slm9* alleviates cell cycle prolongation at the G2 phase caused by Cts1-H359A mutation. 

## 3. Discussion

The presence of cytoophidia in a wide range of species, from prokaryotes to eukaryotes, suggests that it is likely serving an important biological function. Although it has been observed in highly proliferative cells, such as human hepatocellular carcinoma and mouse thymus T cells [5,10], indicating its involvement in cell proliferation, the exact role of cytoophidia remains enigmatic. Therefore, we aimed to investigate the impact of cytoophidia on cell proliferation and elucidate its underlying mechanisms. 

Studies have demonstrated that substitution of the wild-type CTPS protein with a mutant form, in which H^355^ is converted to A^355^ in humans and *Drosophila* and H^360^ is converted to A^360^ in budding yeast, leads to a failure in cytoophidia formation without affecting the tetramer structure of CTPS, which is essential for its activity [14,36]. By aligning the amino acid sequences of CTPS proteins from both *S. cerevisiae* and *S. pombe*, we observed that conversion of amino acid H^359^ to A^359^ abolished cytoophidia formation in fission yeast, as expected.

In this study, using *S. pombe* as a model organism, we demonstrate that loss-filament mutants of CTPS cause a delay in the G2 phase of the cell cycle and result in enlarged cell size during *S. pombe* proliferation. These findings are consistent with previous reports in budding yeast where dysregulated assembly of CTPS leads to impaired growth [24]. Furthermore, expression levels of various genes associated with G2/M transition during cell proliferation were found to be decreased in the Cts1-H359A strain. Our findings indicate that cytoophidia play a role in regulating cell proliferation. 

Previous studies have shown that cytoophidia stabilize the CTPS protein by prolonging its half-life; specifically, the H355A mutant reduced CTPS protein levels in human cancer cells and *Drosophila* adipocytes [18,20]. In our study, we observed a slight decrease in CTPS protein levels in the Cts1-H359A strain, similar to what was observed with the H355A mutant in human cells and *Drosophila* adipocytes. Interestingly, we found that both knockdown and overexpression of CTPS protein influenced cytoophidia formation. These findings suggest mutual regulation between cytoophidia and CTPS protein levels within cells.

Our previous study showed that histone transcription regulator Slm9 is critical for cytoophidia biogenesis. Knockout of *slm9* resulted in a decrease in the percentage of cells containing cytoophidia and mitotic cells while increasing the length of both cytoophidia and cells in *S. pombe* [35]. In our study, Cts1-H359A mutation reduced expression levels of *slm9*, leading to elongation of cell length and delayed cell cycle progression. The observed phenotypes were similar to those observed in *slm9* knockout strains, suggesting a mutual regulation between *slm9* and cytoophidia. 

**Figure 8:**
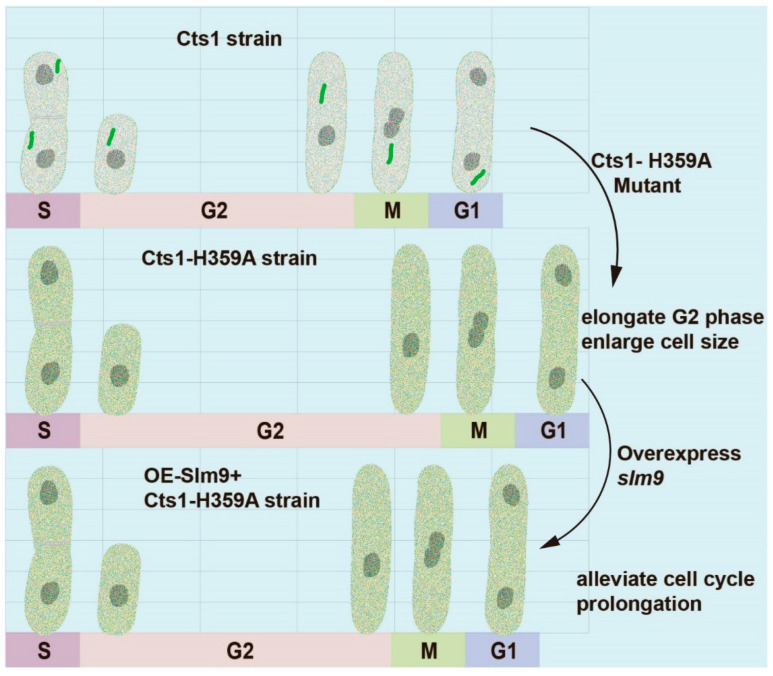
Schematic model for the effect of cytoophidia on cell proliferation. Cts1-H359A mutation prolongs G2 phase and enlarges cell size. Overexpression of *slm9* alleviates cell cycle prolongation in Cts1-H359A strain.

Furthermore, studies have shown that Slm9 regulates Cdc2 activity through Wee1 and that *slm9* deficiency leads to G2 phase delay; immunoaffinity purifications of Slm9 have shown co-purification with Cts1 proteins [33,34]. Our study showed that overexpression of *slm9* partially rescues the prolonged G2 phase induced by the Cts1-H359A mutation (Figure 8). These findings suggest the involvement of Slm9 in the regulation of cytoophidia on cell proliferation.

Since the discovery of cytoophidia in 2010, extensive research has been conducted to elucidate the biological significance of their existence. However, the precise mechanism through which cytoophidia regulate cell proliferation remains elusive. Our investigation may provide valuable insights into unraveling this mechanism. This will be a direction for future exploration. Moreover, further studies are needed to investigate additional biological functions of cytoophidia in other species. 

## 4. Materials and Methods

### 4.1. Yeast Strain and Culture Medium

Fission yeast Cts1-YFP strain was constructed as previously described [7]. Specifically, for the wild-type *S. pombe* strain (h-), ade6-M216, leu1-32, and ura4-D18 were transformed into linearized (SpeI (NEB, Beverly, MA, USA. Cat. R3133S)) pSMUY2-*Ura4*-*cts1*-YFP plasmid. Similarly, the Cts1-H359A-YFP strain was constructed using a PCR-based approach for gene tagging in normal chromosomal locations in fission yeast [37]. Briefly, forward and reverse primers were designed to be complementary to a 20 bp region on each side of the knock-in cassette. Additionally, flanking sequences of 60 bp that were complementary to the immediate 5′-upstream and 3′-downstream endogenous regions of the *cts1* gene were included in each primer (total length of 80–90 bp for each primer). These primers were then used in a PCR reaction with the *cts1*-H359A-YFP-containing plasmid as template to amplify the cassette. The resulting PCR product was purified and approximately 1–2 µg was transformed into fission yeast using the lithium acetate method [37]. The primers used in this study are listed in Table S2. Strains carrying plasmids with overexpressed genes were also prepared using the lithium acetate method. A comprehensive list of all viable *S. pombe* strains can be found in Table S1. Fission yeast was cultured in standard rich media (YES; complete medium enriched with supplements including leucine, uracil, histidine, and adenine at a concentration of 100 µM) or Edinburgh minimal medium 2 (EMM2, thiamine-free) containing 3% glucose and supplemented with necessary amino acids (leucine, uracil, histidine, and adenine at a concentration of 100 µM) for yeast survival at either 30 °C or 32 °C. For plate culture, an additional 2% agar was added and incubated at a temperature of 30 degrees for 3–5 days.

### 4.2. Plasmid Construction

The overexpression plasmids were all constructed using the ClonExpress Ultra One Step Cloning kit (Vazyme, Nanjing, China. Cat. C115-01); genes for the overexpressed plasmids were amplified by PCR from the yeast genome and integrated into corresponding vectors with specific nutritional deficiencies. For example, to construct a plasmid for overexpressing *cts1* or *cts1^H359A^* genes, fission yeast genomic DNA was used as a template for PCR amplification primers with point mutations designed to obtain the *cts1* H359A mutant gene. The *cts1* and *cts1^H359A^* genes were then inserted into leucine selection-labeled overexpression vectors, while *slm9* and *htb1* genes were inserted into adenine-labeled overexpression vectors of fission yeast. All constructed plasmids underwent verification of their DNA sequences by Sanger sequencing. 

### 4.3. Gene Suppression

The *cts1* gene was knocked down using dcas9-mediated CRISPRi. The plasmid pAH237, generously provided by Prof. Tomoyasu Sugiyama, harbors the coding sequence of Cas9 along with a cloning site for the sgRNA and a leucine genetic screening marker. Subsequently, modifications were made to this plasmid to enable the expression of catalytically inactive dCas9 [30]. Briefly, dCas9 eliminates DNA endonuclease activity through mutations D10A and H840A of cas9. The resulting plasmid, which expresses sgRNA and dCas9, is named pdCas9. Targeting sequences (sgRNA) were designed using https://crispr.dbcls.jp/ (accessed on 30 May 2023), which searched for specific 20 bp targeting sequences adjacent to PAM sequences (5′-NGG-3′) in the *S. pombe* genome [38]. The constructed sgRNA-pdCas9 plasmids were transferred into fission yeast using the lithium acetate method. Transcription of the dCas9 gene is controlled by an inducible promoter, nmt1-41p, whose transcription is inhibited by 15 µM thiamine in the culture medium. Therefore, fission yeast cells carrying pdCas9 plasmid were grown on the EMM2 plates supplemented with 20 µM thiamine and necessary amino acid for yeast survival. To induce expression of dCas9 in fission yeast, thiamine was removed by washing the cells with sterilized water twice. Cells were then cultured in thiamine-free EMM2 medium at 32 °C for approximately 20 h with shaking. A list of all sgRNA oligonucleotides and primers used in dcas9-mediated CRISPRi can be found in Table S2.

### 4.4. Growth Assays

Cells were cultured in YES or EMM2 supplemented with necessary amino acids for at least two passages to maintain high proliferative activity. The growth curve was plotted by measuring OD_600_ after reviving cells to a robust growth state and diluting them to an initial OD_600_ of ~0.05 or lower concentrations, such as 0.005. Optical density was measured at corresponding time points during culture. For cell cycle synchronization and release, hydroxyurea (HU) was added to exponentially growing fission yeast at a final concentration of 12 mM, incubated for 4 h, and removed by centrifugation (1000× *g* for 5 min) at room temperature. The collected sample was washed twice and cultured for 3 h to release cells from synchronization. All cells were cultured at 32 °C.

### 4.5. Cell Fixation, Image Acquisition, and Live-Cell Imaging

For the experiment requiring fixed samples, fission yeast cells were collected during either the exponential stage or starvation stage and fixed in 4% paraformaldehyde for 10 min at a temperature of either 30 °C or 32 °C with shaking at 250 rpm. The fixed cells were then washed with PBS and mixed, at a ratio of 2:1, with 1.5% agarose before being dropped onto a glass slide. After covering the sample with a coverslip, the film was sealed using nail polish. Images of the fixed samples were acquired using a Zeiss LSM 980 Airyscan2 microscope (Zeiss, Oberkochen, Germany) equipped with a Plan APO 63x/1.40 OIL objective (Zeiss, Oberkochen, Germany) in Airyscan mode. 

For live imaging, fission yeast cells were cultured in glass-bottomed Petri dishes (Thermo scientificTM, Waltham, MA, USA. Cat. 150460). Specifically, fission yeast was cultured until it reached an exponential state corresponding to an OD_600_ value between 0.5 and 0.8. Then, approximately 0.5 µL of culture was placed at the center of each glass-bottomed Petri dish, followed by gentle addition of dropwise low-melting agarose solution (1.5%) totaling about 400 µL. After allowing it to stand at room temperature for 10 min, the culture medium (about 1 mL) was added. Zeiss Cell Observer SD spinning disk confocal microscope (Zeiss, Oberkochen, Germany) equipped with a 63x OIL objective was used for live-cell imaging at 32 °C.

### 4.6. Western Blotting

Fission yeast protein was extracted using alkaline lysis; subsequently, Western blotting was performed [39]. Briefly, for the protein extraction, 15 mL of exponential stage or starvation stage fission yeast was collected and washed with 1 mL distilled water. The cells were then resuspended in 0.3 mL of distilled water and treated with an equal volume of 0.6 M NaOH for 10 min at room temperature. After centrifugation and removal of the supernatant, the cells were resuspended in 150 µL of 1x SDS sample buffer (ABclonal, Wuhan, China. Cat. RM00001) and incubated at 95 °C for 5 min before being briefly centrifuged to collect the supernatant. An amount of 15 µL of the supernatant was loaded onto a gradient SDS-polyacrylamide gel (GenScript, Nanjing, China. Cat. M00654) and electrophoresed at 120 V in regular Tris-glycine buffer. Protein transfer onto polyvinylidene difluoride membrane was performed using a Trans-blot turbo system (Bio-Rad, California, USA). After blocking with 5% milk, target proteins were detected by using primary antibodies of mouse monoclonal anti-GFP antibody (1: 2000; Roche, Basel, Switzerland. Cat. 11814460001), mouse anti-mCherry (1:5000, Abbkine, Wuhan, China. Cat. Ao2080), and mouse anti-α-Tubulin antibody (1:5000; Sigma, St. Louis, MO, USA. Cat. T6199), followed by secondary antibody Horseradish peroxidase (HRP)-conjugated anti-mouse IgG HRP-linked (1:5000, Cell Signaling, Danvers, MA, USA. Cat. 7076). Non-saturated bands were quantified on ImageJ 1.52i (National Institutes of Health) and presented as a ratio relative to the internal reference α-Tubulin. At least two to three biological replicates were quantified.

### 4.7. RNA Isolation and RT-PCR

Total RNAs were extracted using TransZol Up Plus RNA Kit (TransGen Biotech, Beijing, China. Cat. ER501-01). To disrupt the cell walls of fission yeast, 15 mL log phase cells at the same time point or 5 mL stationary phase cells were collected by centrifugation and resuspended in 1 mL of TransZol Up reagent. Micro glass beads (~200 µL in volume) were added to the suspension, and the cells were then beaten with the glass beads for 10 min on a Vortex machine (30 s of beating followed by 30 s of resting time). Subsequently, the solution was extracted with 200 µL phenol, following the instructions provided in the kit. Equal amounts of RNA templates were reverse transcribed into cDNAs using ABScript III RT Master Mix for qPCR (ABclonal, Wuhan, China. Cat. RK20429). The resulting cDNAs were utilized in qPCR reactions employing Universal SYBR Green Fast qPCR Mix (ABclonal, Wuhan, China. Cat. RK21203) on a BIO-RAD CFX Connect Real-Time System according to the manufacturer’s protocol. Relative expression levels were determined using the comparative CT method with normalization against 18s rRNA as an internal control. The primers used for RT-PCR are listed in Table S2.

### 4.8. Cell Cycle and Cell Size Analysis

ImageJ was utilized for the quantification of cell size, specifically by measuring the cell length of fission yeast. The determination of cell cycle duration was manually performed using ZEN 3.2 (blue edition) software, which facilitated live-cell imaging data analysis and recording of the time required for cell division. Subsequently, statistical analysis was conducted to assess the significance of differences in both cell cycle duration and cell length between various groups, employing one-way ANOVA or unpaired student’s *t*-test.

### 4.9. Quantification and Statistical Analysis

The sample size for each figure is indicated in the figure legends. Statistical significance between conditions was assessed using unpaired Student’s *t*-test. For multiple group comparisons, one-way ANOVA analysis was performed. All statistical analyses were performed in GraphPad Prism, all error bars represent the mean ± standard errors (S.E.M.), and significance is denoted as * *p* < 0.05, ** *p* < 0.01, *** *p* < 0.001 and **** *p* < 0.0001; ns denotes not significant.

## Data Availability

Data is contained within the article and supplementary material.

## Supplementary Materials

The following supporting information can be downloaded at: https://www.mdpi.com/article/10.3390/ijms25010608/s1.
