# Supplementary material for: Cytoophidia Influence Cell Cycle and Size in Schizosaccharomyces pombe
Source: Int J Mol Sci. 2024 Jan 3;25(1):608. doi: 10.3390/ijms25010608 (PMC10779087; doi:10.3390/ijms25010608)
Supplement: Supplementary file 1 [file ijms-25-00608-s001.zip › ijms-2690219-supplementary.pdf]

Cytoophidia Influence Cell Cycle and Size in *Schizosaccharomyces pombe*

Ruolan Deng [1,2,3], Yi-Lan Li [1,2,3] and Ji-Long Liu [1,4,*]

[1] School of Life Science and Technology, ShanghaiTech University, Shanghai 201210, China
[2] CAS Center for Excellence in Molecular Cell Science, Shanghai Institute of Biochemistry and Cell Biology, Chinese Academy of Sciences, Shanghai 200031, China
[3] University of Chinese Academy of Sciences, Beijing 100049, China
[4] Department of Physiology, Anatomy and Genetics, University of Oxford, Oxford OX1 3PT, UK
[*] Correspondence: liujl3@shanghaitech.edu.cn

Supplementary information

Supplementary Tables S1, S2

Supplementary Figures S1-S5

Supplementary Tables S1, S2

Supplementary Table S1. *S. pombe* strains.

Fission Yeast Strain	Genotype
JLL003S	*h- his*-D1 *ura4*-D18 *leu1*-32 *ade6*-M216
JLL005S	*h- cts1*-YFP (ura4+) *ura4*-D18 *leu1*-32 *ade6*-M216
JLL132R	*h- cts1*-H359A-YFP (ura4+) *ura4*-D18 *leu1*-32 *ade6*-M216
JLL127R	*h- cts1*-YFP (leu2+) *ura4*-D18 *leu1*-32 *ade6*-M216
JLL128R	*h- cts1*-H359A-YFP (leu2+) *ura4*-D18 *leu1*-32 *ade6*-M216
JLL122R	*h- cts1*-YFP (ura4+) *ura4*-D18 *leu1*-32 *ade6*-M216/ *ade6*$^{+}$-*htb1*-mCherry
JLL123R	*h- cts1*-H359A-YFP (ura4+) *ura4*-D18 *leu1*-32 *ade6*-M216/ *ade6*$^{+}$-*htb1*-mCherry
JLL125R	*h- cts1*- YFP (ura4+) *ura4*-D18 *leu1*-32 *ade6*-M216/ *leu*$^{+}$-*cts1*-mCherry
JLL126R	*h- cts1*-YFP (ura4+) *ura4*-D18 *leu1*-32 *ade6*-M216/ *leu1*$^{+}$-*cts1*-H359A-mCherry
JLL129R	*h- cts1*- YFP (ura4+) *ura4*-D18 *leu1*-32 *ade6*-M216/ *leu*$^{+}$-mCherry
JLL134R	*h- cts1*-H359A-YFP (ura4+) *ura4*-D18 *leu1*-32 *ade6*-M216/ *ade6*$^{+}$-*slm9*-mCherry
JLL136R	*h- cts1*-H359A-YFP (ura4+) *ura4*-D18 *leu1*-32 *ade6*-M216/ *ade6*$^{+}$-mCherry
JLL135R	*h- cts1*-YFP (ura4+) *ura4*-D18 *leu1*-32 *ade6*-M216/ *ade6*$^{+}$-mCherry

Supplementary Table S2. List of primers.

Primer Name	Sequence (5’-3’)	Use
Ha-Cts1-59F	CGGCTCTCGCGGTGTCGAAGGGATGATTGC CGCAGCCAAGTGGGCTCGTGAAAATAATAC ACCCTACTTGGGCATTTGCCTTGGTATGC	Construction of H359A strain
Ha-ura4-55R	CTATAGATAAAGAAAAACACACCAACAAAC ACACATTATTTCCTAATTCCCGGAATCCCATA TAATTAATGCTGAGAAAGTCTTTGCTG	Construction of H359A strain
Cts1-check-58F	GTCACCATCGTCCTCGTAGG	Verification of H359A strain
YFP-check-59R	GACCATGTGATCGCGCTTCT	Verification of H359A strain
KI864	GCAGCCGACAAGAAGTACAGCATCGGCCTG GCAATCGGCACCAACTCTGTG	pdCas9 construction (D10A mutation)
KI865	GTCGTCCTTCAGAAAGCTCTGAGGCACGAT TGCGTCCACATCGTAGTCGGA	pdCas9 construction (H840A mutation)
KI866	ATCGTGCCTCAGAGCTTTCTGAA	pdCas9 construction (vector region)
KI867	CAGGCCGATGCTGTACTTCTTGT	pdCas9 construction (vector region)
cts1 sgRNA1-F	cacc AATTTTTCTCTCCTCCACGT	Targeting sequence for *cts1* Knock down
cts1 sgRNA1-R	aaac ACGTGGAGGAGAGAAAAATT	Targeting sequence for *cts1* Knock down
cts1 sgRNA2-F	cacc CCTCCACGTACCACCAACTT	Targeting sequence for *cts1* Knock down
cts1 sgRNA2-R	aaac AAGTTGGTGGTACGTGGAGG	Targeting sequence for *cts1* Knock down
cts1 sgRNA3-F	cacc ATATGAAGTACGTTTTAGTA	Targeting sequence for *cts1* Knock down
cts1 sgRNA3-R	aaac TACTAAAACGTACTTCATAT	Targeting sequence for *cts1* Knock down
cts1 sgRNA4-F	cacc GTATCTGGAGGTGTTATTAG	Targeting sequence for *cts1* Knock down
cts1 sgRNA4-R	aaac CTAATAACACCTCCAGATAC	Targeting sequence for *cts1* Knock down
cts1 sgRNA5-F	cacc GGTGTAATTGCCTCTTCCAC	Targeting sequence for *cts1* Knock down
cts1 sgRNA5-R	aaac GTGGAAGAGGCAATTACACC	Targeting sequence for *cts1* Knock down
cas9-r1-58R	TCAGGGCAATCAGGTTTCCG	Verification of pdcas9 expression
nmt1-pro-57F	TCTCACTTTCTGACTTATAGTCGCT	Verification of pdcas9

		expression
sp-cst1-F	ATGATGGCGGTGAAGTCGAT	qPCR for *cts1* transcription analysis
sp-cst1-R	ATAATCACCGCGACGTTCCT	qPCR for *cts1* transcription analysis
sp-slm9-F	TCACCTACACAAGGGCAACC	qPCR for *slm9* transcription analysis
sp-slm9-R	GTTGGGTGAGGAACAGGAGG	qPCR for *slm9* transcription analysis
sp-cdc25-F	ACAAGCTACCCGCCATTTGA	qPCR for *cdc25* transcription analysis
sp-cdc25-R	AGCAACCTTGCCCATCGTAA	qPCR for *cdc25* transcription analysis
sp-cdr1-F	TCGAGTACGTCCCTGATGGT	qPCR for *cdr1* transcription analysis
sp-cdr1-R	GCGATGACAATGCGCTACAG	qPCR for *cdr1* transcription analysis
sp-cdr2-F	AGCTTTCCGGCTCACCTTTT	qPCR for *cdr2* transcription analysis
sp-cdr2-R	CCGATACCAAGAAGCTGCCA	qPCR for *cdr2* transcription analysis
sp-wee1-F	TTATCGACTTCCACACCCGC	qPCR for *wee1* transcription analysis
sp-wee1-R	GTAAGTGGACGAGCGGTAGG	qPCR for *wee1* transcription analysis
sp-sty1-F	TCTTGCGAGGGCTCAAGTTT	qPCR for *sty1* transcription analysis
sp-sty1-R	GAGCCAAACCGAAATCGCAG	qPCR for *sty1* transcription analysis
sp-pom1-F	AGCCGTCGTCCTTTTAGCAA	qPCR for *pom1* transcription analysis
sp-pom1-R	GGCGCAGTGTTAGGTCTTCT	qPCR for *pom1* transcription analysis
sp-tea1-F	GTCACGCAGCTTCTGTTGTG	qPCR for *tea1* transcription analysis
sp-tea1-R	GTGAGGGAGTGAACGGAAGG	qPCR for *tea1* transcription analysis
sp-blt1-F	ACGGTTCATCCATCGTTTTGT	qPCR for *blt1* transcription analysis
sp-blt1-R	GAGTAGACTGGGCGTTTTCGT	qPCR for *blt1* transcription analysis
sp-fin1-F	GAAAATGCGCCTGCTTGTGA	qPCR for *fin1* transcription analysis

sp-fin1-R	CTGTAAGGGCTGCTCCGAAT	qPCR for *fin1* transcription analysis
sp-pop3-F	CAACATGCGGACTCTCAGGT	qPCR for *pop3* transcription analysis
sp-pop3-R	GTTCCATCCTCGCTCGATGT	qPCR for *pop3* transcription analysis
sp-cdc13-F	TCTCGTTTCCGACTGCTTCC	qPCR for *cdc13* transcription analysis
sp-cdc13-R	GAAGGGCACATCACCTCCTC	qPCR for *cdc13* transcription analysis
sp-tor1-F	GATGCCGCTGTAGGTATGCT	qPCR for *tor1* transcription analysis
sp-tor1-R	TGATCCCAGTCACCCAAAGC	qPCR for *tor1* transcription analysis
sp-tor2-F	AACTCCAACAACCAGACGCA	qPCR for *tor2* transcription analysis
sp-tor2-R	ATCCCATTCACCCAAAGCGT	qPCR for *tor2* transcription analysis
taf10-F	GGAAGAAGAGGTGACCGTGG	qPCR for *taf10* transcription analysis
taf10-R	AACGGAAGAAATCGGGCCTT	qPCR for *taf10* transcription analysis
18s rRNA-F	TGCCCGAGCTTCTAAGTGTG	qPCR for *18s rRNA* transcription analysis
18s rRNA-R	ACCCCATCCTGACCCCATTA	qPCR for *18s rRNA* transcription analysis
gpd3-F	TGCCTACACTGCTTCCAAGG	qPCR for *gpd3* transcription analysis
gpd3-R	CCCAAAACCGAACGCATGTT	qPCR for *gpd3* transcription analysis
nda2-F	ACAATTACGCCCGTGGACAT	qPCR for *nda2* transcription analysis
nda2-R	TGTTCAAGCGCTCCAGAAGT	qPCR for *nda2* transcription analysis
pda1-F	CCCACCATAACGGTGCTACA	qPCR for *pda1* transcription analysis
pda2-R	AGCAAACAAGTCAATCAAGCGT	qPCR for *pda1* transcription analysis
tdh1-F	GTGTCGTTGACTTGGTTGCC	qPCR for *tdh1* transcription analysis
tdh1-R	CACCCACCCGACGTTTCTTA	qPCR for *tdh1* transcription analysis
actin-F	GGTCAAGATTGTTGCTCCTC	qPCR for *actin*

		transcription analysis
actin-R	CGCTCTCATCATACTCTTGC	qPCR for *actin* transcription analysis

Supplementary Figures S1-S4

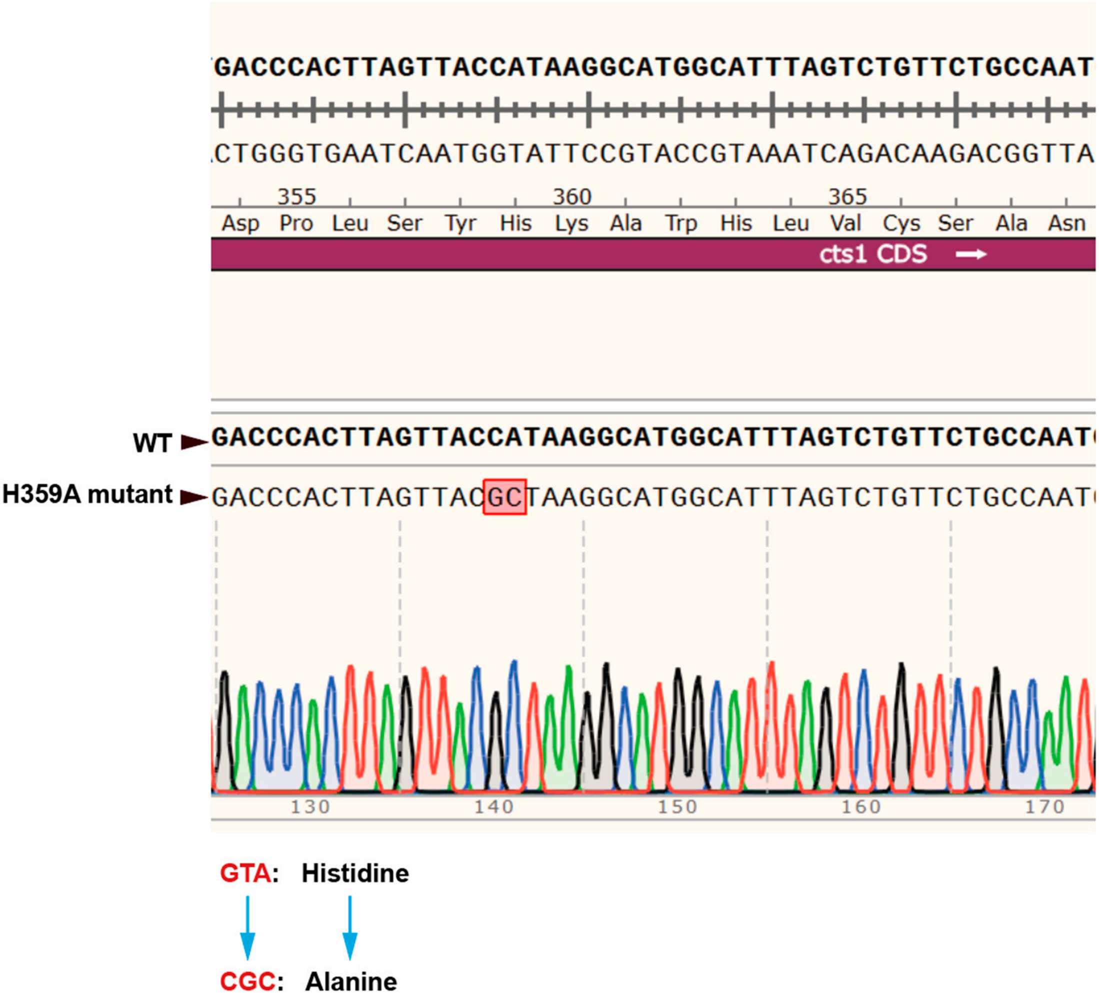

Supplemental Figure S1. Sequencing analysis.

Sequencing analysis of genomic DNA to confirm CTPS H359A mutation in Cts1-H359A strain.

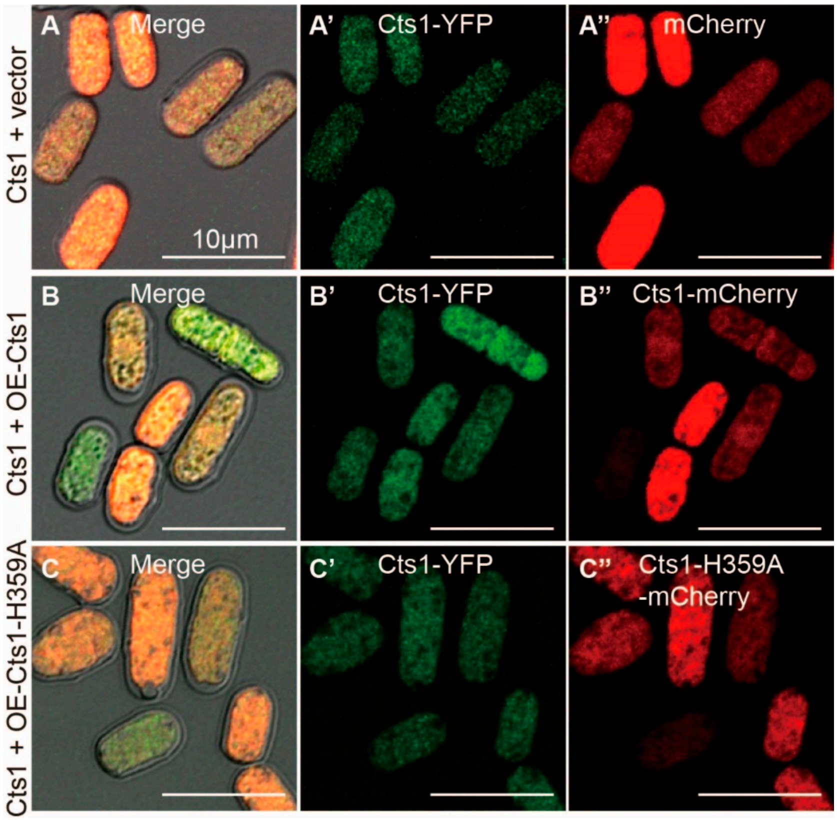

Supplemental Figure S2. Cytoophidia do not form at stationary phase in various fission yeast strains. (A-C") Photos of Cts1 strains that overexpress mCherry (A-A"), CTPS-mCherry (OE-Cts1, B-B"), and Cts1-H359A-mCherry (OE-Cts1-H359A, C-C") at the stationary phase. Channels: green (CTPS), red (mCherry) and bright field. Scale bars, 10 μm.

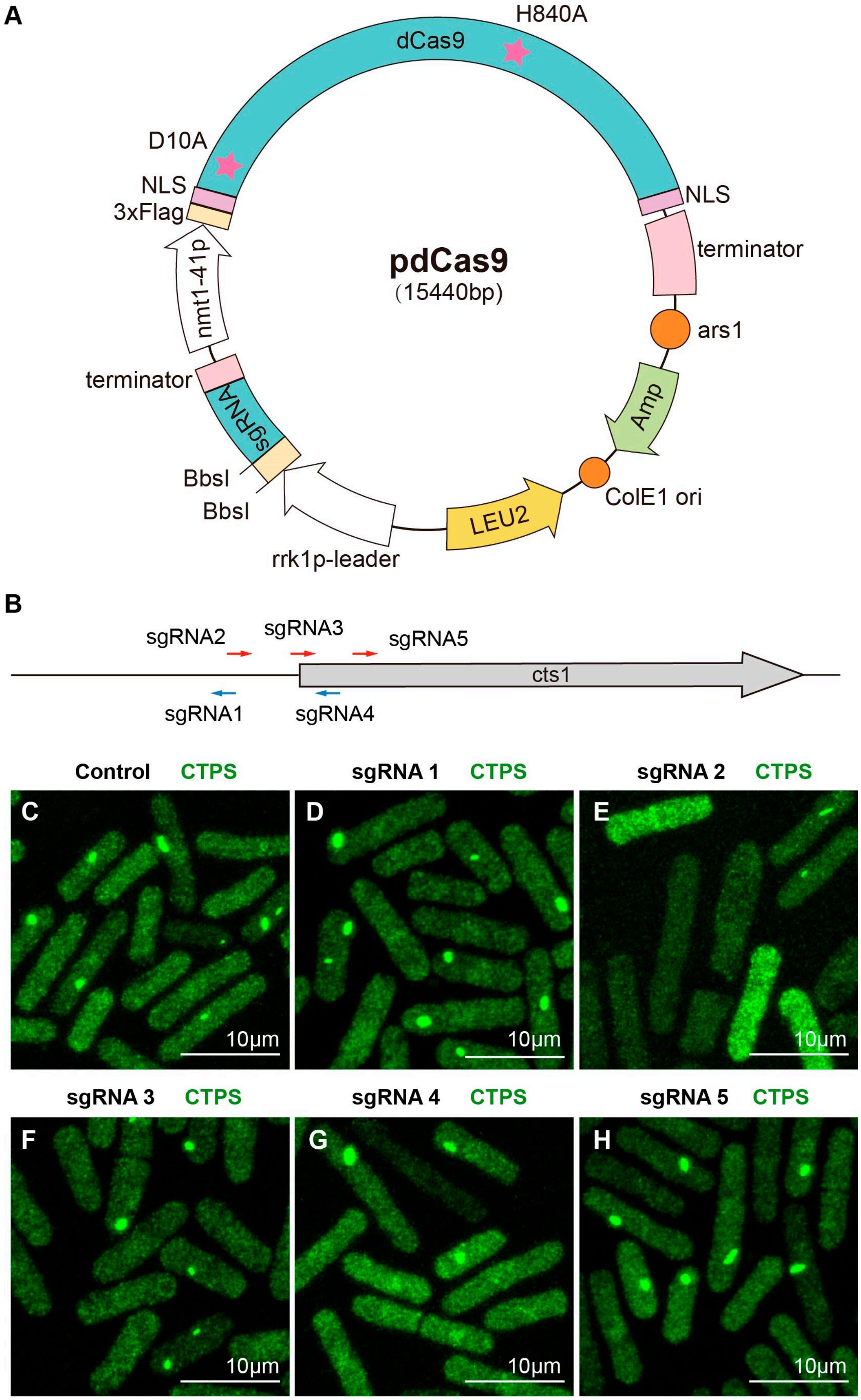
A
H840A
dCas9
D10A
NLS
3xFlag
nmt1-41p
NLS
terminator
pdCas9
(15440bp)
ars1
terminator
sgRNA
Amp
BbsI
BbsI
ColE1 ori
LEU2
rrk1p-leader
B
sgRNA2
sgRNA3
sgRNA5
cts1
sgRNA1
sgRNA4
Control CTPS
sgRNA 1 CTPS
sgRNA 2 CTPS
C
D
E
10μm
10μm
10μm
sgRNA 3 CTPS
sgRNA 4 CTPS
sgRNA 5 CTPS
F
G
H
10μm
10μm
10μm

Supplemental Figure S3. knockdown the CTPS by using the CRISPR-dCas9 system. (A) Plasmid map of pdCas9. dCas9, humanized dCas9, originally from *Streptococcus pyogenes*. The pentagram indicated mutations introduced in the dCas9 gene to cause the indicated amino acid substitutions. nmt1-41p, a moderate-strength variant of the nmt1 promoter, the transcription of which is inducible. When the targeting sequence is not replaced, pdCas9 expresses a sgRNA with a nonsense targeting sequence in fission yeast. **(B)** Diagram of the designed targeting sequences with the *cts1* gene. Red and blue arrows indicate targeting sequences of sgRNAs that base-pair with the template and non-template strand of the targeted DNA, respectively. **(C)** Photographs of the CTPS-YFP strains which expressed the pdCas9 plasmid carried nonsense sgRNA (as a control group), sgRNA1, sgRNAs2, sgRAN3, sgRAN4, sgRAN5, respectively, at log phase. Scale bars, 10 μm.

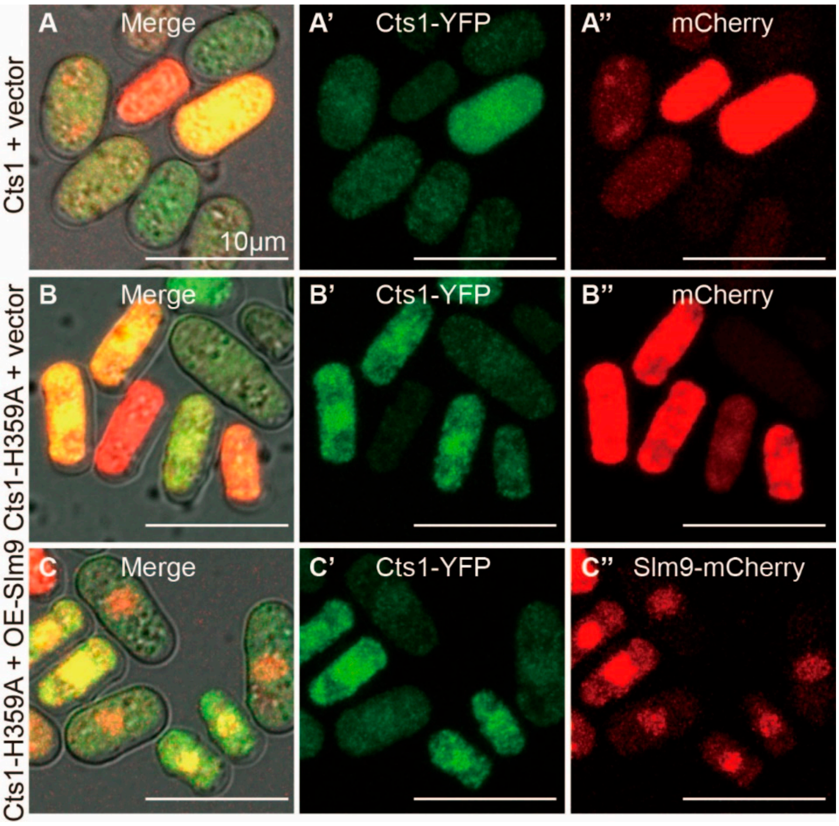

Supplemental Figure S4. Overexpression of *slm9* in Cts1-H359A strain. **(A-A")** Photographs of Cts1 strain that overexpressed only mCherry at the stationary phase. Scale bars, 10 µm. **(B-B")** Photographs of Cts1-H359A strain that overexpressed only mCherry at the stationary phase. Scale bars, 10 µm. **(C-C")** Photographs of Cts1-H359A strain that overexpressed Slm9-mCherry at the stationary phase. Scale bars, 10 µm. Channels: green (CTPS), red (mCherry) and bright field.

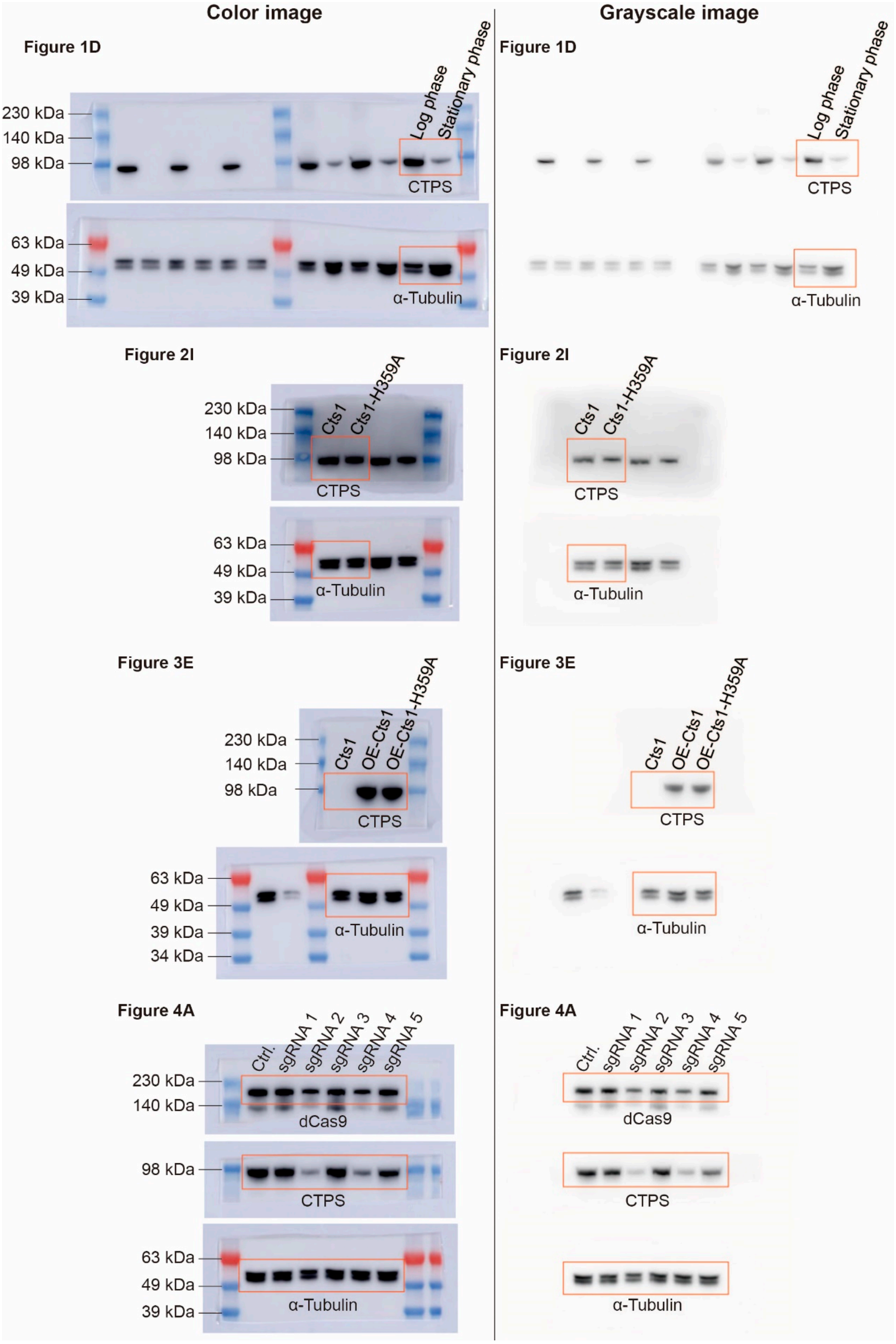

Supplemental Figure S5. Uncropped original Western Blotting images in figure 1D, 2I, 3E, 4A.
